# Bilateral morphea en coup de sabre following onabotulinum toxin A injections

**DOI:** 10.1016/j.jdcr.2025.06.053

**Published:** 2025-08-05

**Authors:** Olamide Adebayo, Celestina Okoye, Jennifer L. Shastry

**Affiliations:** a Feinberg School of Medicine, Northwestern University, Chicago, Illinois; b Department of Dermatology, Northwestern Medicine Chicago, Illinois

**Keywords:** bilateral morphea, en coup de sabre, onabotulinum toxin A

## Introduction

Morphea en coup de sabre, a subtype of linear morphea, is an inflammatory disorder of the skin that can affect underlying soft tissue, causing cosmetic and functional impairment.^1^ This condition typically appears unilaterally on the frontoparietal scalp and forehead with band-like indurated skin lesions.^1^^,^^2^ Bilateral involvement is rare, with onset usually occurring in pediatric cases.^1^ This report aims to describe a unique case of diagnosed bilateral morphea en coup de sabre that developed following onabotulinum toxin A (BTXA) injections.

## Case report

**Fig 1:**
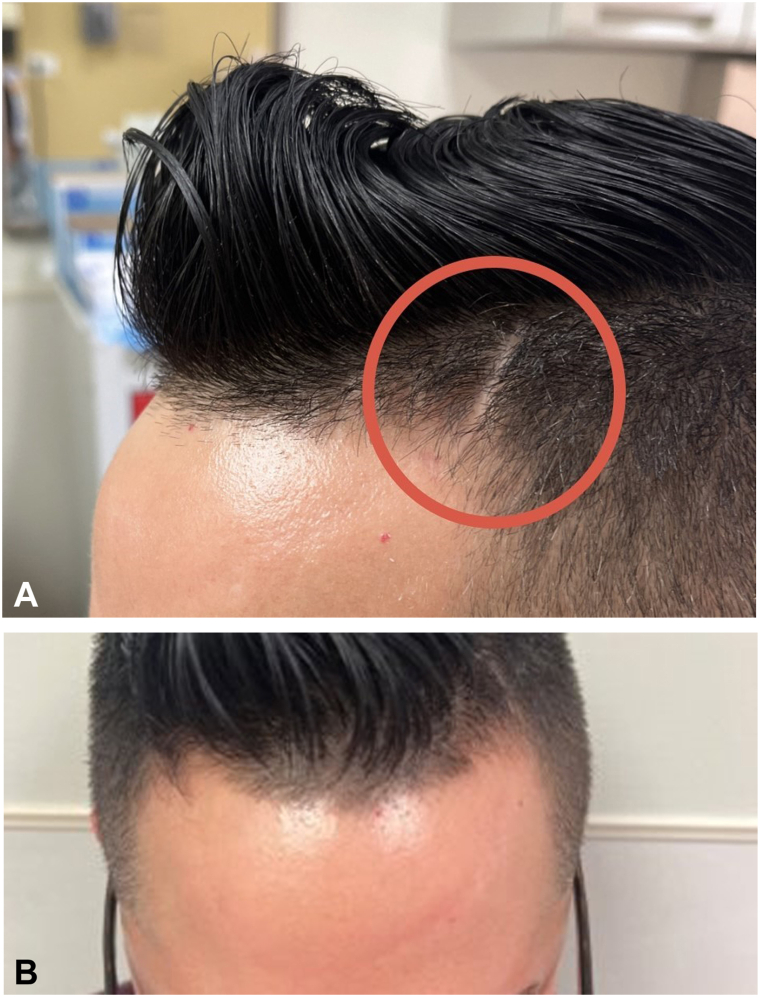
**A,** Atrophic plaque of left frontal scalp, present since childhood. **B,** Patient presenting with an arcuate firm atrophic plaque of the bilateral forehead extending into the left frontal scalp.

A 37-year-old man presented to our institution with a progressive indentation on his forehead, which began following BTXA injections by an outside dermatologist. The patient noted having an indentation on his left frontal scalp since childhood which was not evaluated by a dermatologist (see Fig 1, *A*). The patient began receiving BTXA injections to the frontalis and glabella in early 2021. He noticed the development of an indentation on the left forehead approximately 3 months after the first injection. He continued receiving neuromodulator injections every 3 months and saw the progression of the indentation to involve both sides of the forehead, ultimately making a U shape. He denied similar indentations or rashes elsewhere on the body. The patient denied experiencing skin tightening elsewhere on the body, Raynaud's phenomenon, shortness of breath, cough, headaches, or a history of seizures but reported fatigue.

On examination, there was an arcuate, firm, atrophic plaque on the bilateral forehead, extending into the left frontal scalp (see Fig 1, *B*). Telangiectasias were present on the bilateral cheeks, temples, and forehead. The remainder of the physical examination was unremarkable.

**Fig 2:**
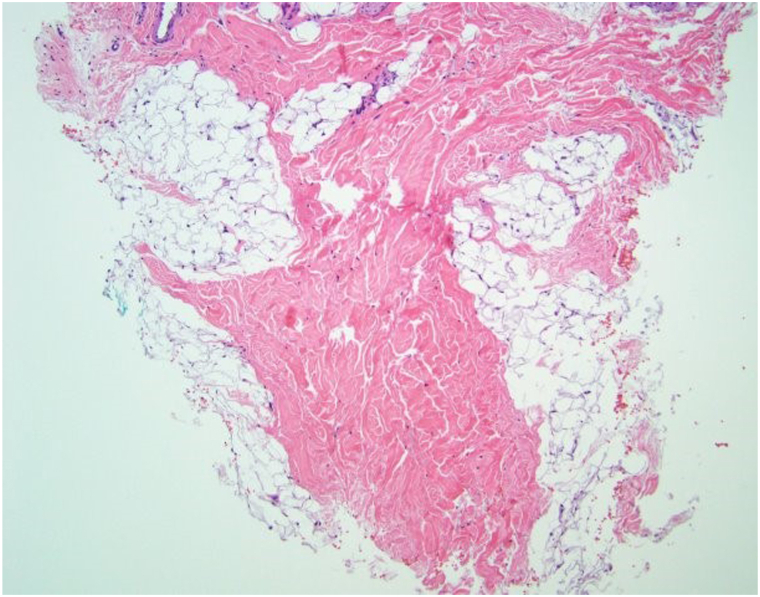
Punch biopsy of the patient's left lateral forehead showing deep dermal sclerosis with extension into adipose tissue with hematoxylin and eosin staining.

Laboratory results revealed elevated antinuclear antibodies with a negative reflex panel (1:320, speckled). Serum inflammatory markers were within normal limits. Magnetic resonance imaging of the brain showed no intracranial abnormalities. Skin biopsy of the left lateral forehead revealed deep dermal sclerosis extending into the adipose tissue, consistent with linear morphea, en coup de sabre variant (see Fig 2).

The patient was initiated on methotrexate (10 mg weekly) and daily folic acid supplementation. Tacrolimus ointment was prescribed for application to the indurated plaques twice daily. The patient was recommended to discontinue BTXA injections.

At a 3-month follow-up, the plaque showed improvement on the bilateral forehead, with no change in the pre-existing scalp lesion or evidence of hemifacial atrophy. Methotrexate was discontinued per patient preference and tacrolimus ointment was continued. At a 6-month follow-up, the patient reported no changes or progression of the linear morphea and denied any pain or itching. Given the stability of the plaques off methotrexate, the patient was cleared to pursue filler injections for cosmetic correction of the morphea if desired.

## Discussion

Morphea en coup de sabre is a rare form of localized scleroderma that usually presents unilaterally and is more prevalent in females. Few cases have described “morphea-like” lesions or depressions following BTXA injections, but never a true morphea diagnosis with biopsy, which is unique to our case.^3^, ^4^, ^5^ Landau et al described 3 cases of morphea-like lesions in women treated at different clinics after aesthetic BTXA injections, with 2 of those cases resolving spontaneously before skin biopsy. The last case's biopsy showed a normal distribution of dermal collagen, adnexal structures, and elastic tissue with no evidence of morphea.^5^ All 3 cases gradually resolved over 3 months.^5^ Similarly, Nyckowski et al described 2 cases of linear cutaneous depressions after BTXA injections, which resolved within 2 to 7 months following normal saline injections.^4^ No biopsies were obtained for these cases due to the gradual improvement of the depressions. In all reported cases, the lesions were unilateral or midline.

It is hypothesized that aberrant muscle contractions may trigger “morphea-like” indentations.^4^ Due to the cutaneous insertions in the frontalis muscle, changes in muscle contractility might result in superficial skin changes, such as increased muscle contraction in depressed areas relative to surrounding skin.^6^^,^^7^ The depressions, however, tend to be superficial and not the result of an inflammatory process.

This case report highlights a distinctive case of the progression of bilateral morphea en coup de sabre in a patient following BTXA injections. While it is not possible to prove causality, the previous cases of the morphea-like lesions post-BTXA and this case of a possible exacerbation of preexisting morphea (affecting the left scalp) in combination yields an interesting context that is helpful for dermatologists to consider when administering cosmetic BTXA in patients. The risk of disease re-activation and progression should be discussed with any patient with en coup de sabre morphea considering cosmetic BTXA. It has been suggested to wait 3-6 months before conducting a biopsy of a cutaneous depression following BTXA injections; however, a detailed skin and medical history should be considered before decision-making. If there is an exacerbation of preexisting morphea, the primary focus should be on slowing or stopping the progression of the disease and improving cosmetic outcomes. First-line therapy to halt progressing morphea involves a combination of methotrexate and oral corticosteroids. Other treatment options include topical steroids, topical calcineurin inhibitors, UVA1 phototherapy in active morphea, and hyaluronic acid fillers for cosmetic improvement of stable disease.^8^

## Conflict of interest

Dr Shastry is an advisory board member for Johnson & Johnson (guselkumab), which is not relevant to this report. Dr Okoye and Author Adebayo have no conflicts of interest to declare.
